# Current Research on HIV Drug Resistance—A Topical Collection with “*Pathogens*”

**DOI:** 10.3390/pathogens11090966

**Published:** 2022-08-25

**Authors:** Hezhao Ji

**Affiliations:** 1 National Microbiology Laboratory at JC Wilt Infectious Diseases Research Centre, Public Health Agency of Canada, Winnipeg, MB R3E 3R2, Canada; hezhao.ji@phac-aspc.gc.ca; 2 Max Rady College of Medicine, University of Manitoba, Winnipeg, MB R3E 0J9, Canada

Viral drug resistance is an everlasting topic for HIV/AIDS professionals from clinical, laboratory and public health perspectives [1]. As one of the most challenging human viral pathogens, HIV is notorious for its significant genetic and antigenic diversity, both intra-host and inter-host, resulting from poor proofreading of the viral reverse transcriptase as the virus replicates coupled with its high replication rate [2,3,4]. Unsurprisingly, HIV drug resistance (HIVDR) was reported soon after the commercialization of the first antiretroviral drug [5]. Since then, HIVDR has been symbiotic with all HIV drugs currently applied in antiretroviral therapy (ART), although the genetic barriers for the resistance development again different drugs vary. *Pathogens* launched a topical collection of submissions in 2021 to catch the latest advances in HIVDR diagnosis, surveillance and research perspectives. While this collection remains open for new submissions, we already have ten excellent articles published thus far. This editorial piece provides a brief walkthrough of these articles and highlights their significant contributions to the HIVDR field in general. 

Next-generation sequencing (NGS)-based HIVDR testing is a trending new standard for HIVDR typing, attributable to its high sensitivity and accuracy in semi-quantitative detection of HIVDR variants, especially those present at lower frequencies [6]. Li et al. applied NGS HIVDR testing in a cross-sectional study in China, revealing that HIVDR prevalence in patients under ART interruption is higher than in ART-naïve patients or those on ART therapy [7]. It provides more convincing evidence, reassuring the improved sensitivity of NGS in detecting lower abundance HIVDR mutations. 

The application of integrase strand transfer inhibitor (INSTI)-based therapy is rising globally in first-line ART, attributable largely to the well-documented higher genetic barriers for resistance development against INSTIs. Two articles in this collection dealt with HIVDR against INSTIs. Seatla K et al. reported their findings in examining the correlation between 3′-polypurine tract (3′PPT) variations in the HIV-1 *nef* gene and failure of INSTI-based ART treatment [8]. Multiple HIV-1 genetic variations outside the *pol* gene have been reported to be associated with HIVDR occurrence, although they do not directly alter the coding of drug-targeted HIV enzymes [9,10,11,12]. One such viral genetic trait identified by in vitro breakthrough selection experiments is the 3′PPT variation in the HIV-1 *nef* gene, reported to be contributing to INSTI resistance [9]. However, several later studies failed to confirm this association in patients failing INSTI-based ART, but all reported the high genetic conservation of this region [13,14,15]. By examining the 3′PPT from 6009 HIV-1 subtype C sequences from Botswana, this study provides solid evidence of the high conservation of the 3′PPT sequence and rules out the causal connection between variations in this motif and INSTI resistance [8]. Martin et al. reported on the coevolution of the ART-targeted HIV-1 genes and the potential impacts of the co-evolved HIV-1 protease (PR) and reverse transcriptase (RT) genes on the HIV-1 viral fitness and its susceptibility to INSTIs [16]. This study highlights the close interactions of the ART-targeted viral enzymes/genes during ART, which necessitate the analysis of the whole HIV-1 *pol* gene, or even the entire genomes, to better decipher the mechanisms of HIVDR occurrence in the context of ART. 

HIVDR occurrence is, by all means, a multifactorial phenomenon involving many interconnected factors from social, economic, medical and behavioural perspectives. Kiekens et al. developed and presented a comprehensive local systems map that enables in-depth analysis and an understanding of HIVDR-relevant factors in a complex adaptive system [17]. While it was developed in a Tanzania-based study, this system could be easily adapted or adopted in other settings to better understand the local HIVDR situation and identify actionable strategies to combat HIVDR. 

Population-level HIVDR surveillance provides valuable information in monitoring the HIVDR situation in the region, evaluating the impact of HIVDR-related policies and strategies and forming treatment guidelines to optimize clinical outcomes at population levels [1,18]. Two HIVDR surveillance studies from Mexico were included in this topical collection. García-Morales et al. presented a four-year observational study monitoring the pre-treatment HIVDR prevalence trend against protease inhibitor (PI), RT inhibitor (RTI) and INSTI in a large patient cohort from Mexico City during 2017~2020 [19]. Caro-Vega et al. presented a report describing the clinical outcomes of participants from a 2017 to 2018 national representative HIV PDR survey in Mexico [20]. Both articles exemplify large-scale HIVDR monitoring for a designated population or patient cohort and for the evaluation of its clinical significance. 

Following the above is an excellent report on the spectrum of atazanavir-selected PI resistance mutation from Rhee et al. [21]. While ritonavir-boosted atazanavir is now an often-used second-line PI option in ART, especially in low- to middle-income countries (LMIC), there is a paucity of studies examining the PR mutations occurring in patients receiving atazanavir treatment. To fill this gap, the authors analyzed 1497 PR sequences from patients receiving boosted or unboosted atazanavir treatments and profiled all PR mutations selected by atazanavir in previously PI-naïve patients who failed atazanavir-containing regimens. I highly recommend this article for HIVDR researchers and clinicians in order to better understand the cross-resistance among commonly applied PIs for the optimal use of these drugs in clinical settings. 

The last group of manuscripts in this collection includes three review or commentary articles summarizing the advances in three perspectives pertaining to HIVDR laboratory testing. Munyuza et al. reviewed the recent progress in applying a probe-capturing enrichment strategy for improved viral template recovery from samples containing degraded viral RNA/DNA or low viral loads for HIV and HCV genotyping [22]. Chua et al. summarized up-to-date advances in point-of-care test (POCT) technologies that may help boost the accessibility and simplicity of HIVDR assays with improved cost-effectiveness [23]. This information is valuable for promoting de-centralized HIVDR testing in LMIC where ART coverage is scaling up while HIVDR monitoring lags behind due to resource limitations. Ji and Sandstrom provided a comprehensive review of all clinical analytes that have been used in HIVDR testing thus far in both research and clinical settings [24]. It may assist in the optimal selection of specimens for different HIVDR testing needs. 

Taken together, I hope this HIVDR topical collection will contribute to further advancements in basic research, laboratory testing and effective management pertaining to HIVDR. New submissions are always welcome when the collection is still open. 

As the collection editor, I appreciate the collective efforts from all authors, reviewers, and editorial personnel of *Pathogens* who have made this topical collection a reality. Thank you!

## References

[1] World Health Organization (2017). Global Action Plan on HIV Drug Resistance 2017–2021.

[2] Coffin J.M. (1995). HIV population dynamics in vivo: Implications for genetic variation, pathogenesis, and therapy. Science.

[3] Ho D.D. (1997). Perspectives series: Host/pathogen interactions. Dynamics of HIV-1 replication in vivo. J. Clin. Investig..

[4] Menendez-Arias L. (2002). Targeting HIV: Antiretroviral therapy and development of drug resistance. Trends Pharmacol. Sci..

[5] Larder B.A., Darby G., Richman D.D. (1989). HIV with reduced sensitivity to zidovudine (AZT) isolated during prolonged therapy. Science.

[6] Ji H., Sandstrom P., Paredes R., Harrigan P.R., Brumme C.J., Avila R.S., Noguera-Julian M., Parkin N., Kantor R. (2020). Are We Ready for NGS HIV Drug Resistance Testing? The Second “Winnipeg Consensus” Symposium. Viruses.

[7] Li M., Liang S., Zhou C., Chen M., Liang S., Liu C., Zuo Z., Liu L., Feng Y., Song C. (2021). HIV Drug Resistance Mutations Detection by Next-Generation Sequencing during Antiretroviral Therapy Interruption in China. Pathogens.

[8] Seatla K.K., Maruapula D., Choga W.T., Morerinyane O., Lockman S., Novitsky V., Kasvosve I., Moyo S., Gaseitsiwe S. (2021). Limited HIV-1 Subtype C nef 3′PPT Variation in Combination Antiretroviral Therapy Naive and Experienced People Living with HIV in Botswana. Pathogens.

[9] Malet I., Subra F., Charpentier C., Collin G., Descamps D., Calvez V., Marcelin A.G., Delelis O. (2017). Mutations Located outside the Integrase Gene Can Confer Resistance to HIV-1 Integrase Strand Transfer Inhibitors. mBio.

[10] Dicker I.B., Samanta H.K., Li Z., Hong Y., Tian Y., Banville J., Remillard R.R., Walker M.A., Langley D.R., Krystal M. (2007). Changes to the HIV long terminal repeat and to HIV integrase differentially impact HIV integrase assembly, activity, and the binding of strand transfer inhibitors. J. Biol. Chem..

[11] Rabi S.A., Laird G.M., Durand C.M., Laskey S., Shan L., Bailey J.R., Chioma S., Moore R.D., Siliciano R.F. (2013). Multi-step inhibition explains HIV-1 protease inhibitor pharmacodynamics and resistance. J. Clin. Investig..

[12] Fun A., Wensing A.M., Verheyen J., Nijhuis M. (2012). Human Immunodeficiency Virus Gag and protease: Partners in resistance. Retrovirology.

[13] Malet I., Delelis O., Nguyen T., Leducq V., Abdi B., Morand-Joubert L., Calvez V., Marcelin A.G. (2019). Variability of the HIV-1 3′ polypurine tract (3′PPT) region and implication in integrase inhibitor resistance. J. Antimicrob. Chemother..

[14] Wei Y., Sluis-Cremer N. (2021). Mutations in the HIV-1 3′-Polypurine Tract and Integrase Strand Transfer Inhibitor Resistance. Antimicrob. Agents Chemother..

[15] Acharya A., Tagny C.T., Mbanya D., Fonsah J.Y., Nchindap E., Kenmogne L., Jihyun M., Njamnshi A.K., Kanmogne G.D. (2020). Variability in HIV-1 Integrase Gene and 3′-Polypurine Tract Sequences in Cameroon Clinical Isolates, and Implications for Integrase Inhibitors Efficacy. Int. J. Mol. Sci..

[16] Martin S.A., Cane P.A., Pillay D., Mbisa J.L. (2021). Coevolved Multidrug-Resistant HIV-1 Protease and Reverse Transcriptase Influences Integrase Drug Susceptibility and Replication Fitness. Pathogens.

[17] Kiekens A., Mosha I.H., Zlatic L., Bwire G.M., Mangara A., de Dierckx C.B., Decouttere C., Vandaele N., Sangeda R.Z., Swalehe O. (2021). Factors Associated with HIV Drug Resistance in Dar es Salaam, Tanzania: Analysis of a Complex Adaptive System. Pathogens.

[18] World Health Organization (2021). HIV Drug Resistance Report 2021.

[19] Garcia-Morales C., Tapia-Trejo D., Matias-Florentino M., Quiroz-Morales V.S., Davila-Conn V., Beristain-Barreda A., Cardenas-Sandoval M., Becerril-Rodriguez M., Iracheta-Hernandez P., Macias-Gonzalez I. (2021). HIV Pretreatment Drug Resistance Trends in Mexico City, 2017–2020. Pathogens.

[20] Caro-Vega Y., Alarid-Escudero F., Enns E.A., Sosa-Rubi S., Chivardi C., Pineirua-Menendez A., Garcia-Morales C., Reyes-Teran G., Sierra-Madero J.G., Avila-Rios S. (2021). Retention in Care, Mortality, Loss-to-Follow-Up, and Viral Suppression among Antiretroviral Treatment-Naive and Experienced Persons Participating in a Nationally Representative HIV Pre-Treatment Drug Resistance Survey in Mexico. Pathogens.

[21] Rhee S.Y., Boehm M., Tarasova O., Di T.G., Abecasis A.B., Sonnerborg A., Bailey A.J., Kireev D., Zazzi M., The EuResist Network Study Group (2022). Spectrum of Atazanavir-Selected Protease Inhibitor-Resistance Mutations. Pathogens.

[22] Munyuza C., Ji H., Lee E.R. (2022). Probe Capture Enrichment Methods for HIV and HCV Genome Sequencing and Drug Resistance Genotyping. Pathogens.

[23] Chua R.J., Capina R., Ji H. (2022). Point-of-Care Tests for HIV Drug Resistance Monitoring: Advances and Potentials. Pathogens.

[24] Ji H., Sandstrom P. (2022). Overview of the Analytes Applied in Genotypic HIV Drug Resistance Testing. Pathogens.

